# Ultrasound cesarean scar assessment one year postpartum in relation to one‐ or two‐layer uterine suture closure

**DOI:** 10.1111/aogs.13714

**Published:** 2019-09-26

**Authors:** Jiri Hanacek, Jiri Vojtech, Iva Urbankova, Michal Krcmar, Petr Křepelka, Jaroslav Feyereisl, Ladislav Krofta

**Affiliations:** ^1^ Institute for the Care of Mother and Child Prague Czech Republic; ^2^ 3rd Medical Faculty Charles University Prague Czech Republic

**Keywords:** cesarean section, double‐layer technique, suture healing, uterine scar, uterine suture

## Abstract

**Introduction:**

This study compared healing of the scars after cesarean section during the first postpartum year using a single‐ or double‐layer suturing technique. Scarring was assessed by a transvaginal ultrasound. We explored the appearance and localization of uterine scars with regard to the obstetric history. Our aim was to compare the position of the scar or defect, if present, its dimensions, and any residual myometrium with respect to the suturing technique during the cesarean section.

**Material and methods:**

Women with uncomplicated singleton pregnancies indicated for elective or acute cesarean section were randomly allocated to the uterine closure technique group. During the first postpartum year, their lower uterine segment was examined with a transvaginal ultrasound in three consecutive visits at 6 weeks, 6 months and 12 months.

**Results:**

324 women attended the 12‐month visit; of these, 149 underwent single‐layer closure of the uterine incision and 175 double‐layer technique. A higher proportion of the defects is seen in the single‐layer closure technique of suturing. Defects in the single‐layer group were wider (0.002) and the residual myometrial thickness in the single‐layer group were thinner (0.019). Women who underwent cesarean section at the stage of full cervical dilation had scars that were closer to the external cervical os (0.000). The position of the uterus varies greatly between controls (0.000). The combination of uterine position and scar defect presence changed significantly between controls (0.001), and was significantly dependent on the suturing method (0.003). Defects with or without contact with the uterine cavity changed statistically between controls (0.017). Both types of defects were more common in the single‐layer closure technique group.

**Conclusions:**

The findings of this study demonstrate that double‐layer technique with the first continuous nonlocking suture followed by a second continuous nonlocking suture is associated with better suture healing and greater residual myometrial thickness. No difference was observed between single‐ and double‐layer closure for the presence of maternal infectious morbidity, wound infection or blood transfusion.

**Abbreviations**
CS: cesarean section
DLT: double‐layer technique
RMT: residual myometrial thickness
SLT: single‐layer technique

**Key message**

Deficient uterine scar healing represents a side effect with potential negative long‐term consequences. This study demonstrates that a double‐layer technique with the first continuous nonlocking suture followed by a second continuous nonlocking suture is associated with better uterine suture healing.

## 1 INTRODUCTION

There are multiple factors driving the increase in cesarean section (CS) rates internationally. Demographic factors in the economically developed world partly explain the rise.^1^, ^2^ Deficient uterine scar healing represents a side effect with negative consequences. Serious obstetric complications may occur in the subsequent pregnancy such as uterine scar dehiscence (0.6‐3.8%), uterine scar rupture (0.2‐3.8%) and cesarean scar pregnancy, which may be associated with morbidly adherent placenta.^3^, ^4^ In the long‐term, women with a scar defect may also suffer from gynecological problems.^5^, ^6^, ^7^

Transvaginal ultrasound is validated tool to evaluate uterine scar defects commonly referred to as “niche”.^8^, ^9^ Healing and scar maturation are influenced by the suture technique, number of previous CS deliveries and patient's medical and obstetric history.^10^ There are many techniques for the closure of the uterine incision. In the short‐term, no clear benefit of any of the randomized comparisons has been shown. The objective of this study was to compare the effect of single‐ vs double‐layer closure technique on uterine scar morphology in primiparas after CS.

## 2 MATERIAL AND METHODS

A prospective randomized study was conducted between November 2011 and September 2014 in the Institute for the Care of Mother and Child in Prague (tertiary perinatological center). The study included a cohort of nulliparous women with a singleton pregnancy who underwent first delivery by any type of CS. Women were invited for three consecutive control visits at 6 weeks, 6 months and 12 months postpartum. Women who did not complete all postoperative visits were excluded from the analysis.

**Figure 1:**
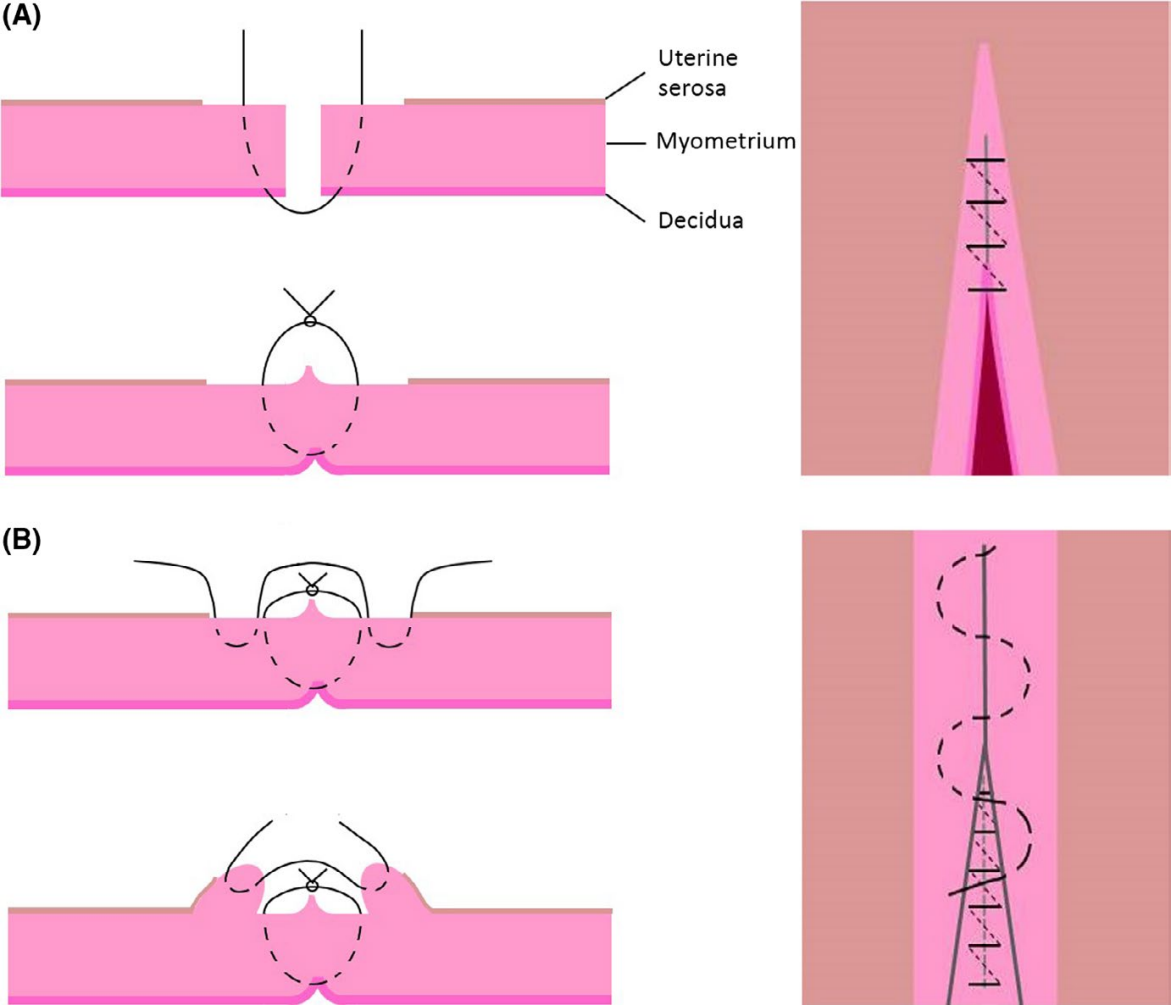
Uterine suture technique. (A) Unlocked single‐layer closure. Decidua was incorporated in the suture. The uterine serosa is not included in the suture. (B) Double‐layer closure. First layer unlocked suture including decidua. Second layer unlocked suture taking superficial part of myometrium. Uterine serosa is not included in the suture [Color figure can be viewed at http://wileyonlinelibrary.com]

The goal of the proposed study is to test the null hypothesis that the mean thickness of the uterine scar in the two groups is equal. Women with a uterine scar defect had thinner full lower uterine segment and thinner myometrial layer. The optimal cut‐off value varied from 2.0 to 3.5 mm for full lower uterine segment thickness and from 1.4 to 2.0 mm for myometrial layer.^11^ We calculated the sample size to observe a difference of 1.0 mm of myometrial thickness between uterine closures. For the comparison of the influence of both surgical techniques on muscle layer thickness we used a two‐tailed test, which means that an effect in either direction will be interpreted. The significance level for a test of the null hypothesis was set at 0.050 (alpha). With the proposed sample size of 103 each for the two groups, the study would have a power of 90.1% to yield a statistically significant result. This computation assumes that the mean difference for myometrial layer thickness is 1.0 mm and the common within‐group standard deviation is 2.2 mm (on the basis of our feasibility studies). This effect was selected as the smallest effect that would be important to detect, in the sense that any smaller effect would not be of clinical or substantive significance. It was also assumed that this effect size was reasonable, in the sense that an effect of this magnitude could be anticipated in this field of research. Women indicated for CS were randomly allocated after opening a consecutively numbered envelope containing the uterine closure technique group, either single‐layer technique (SLT) or double‐layer technique (DLT) closure of the hysterotomy**.** This study represents a subanalysis from a large prospective cohort of healthy women with a first singleton pregnancy who delivered at or beyond 37 weeks. The SLT involved a single continuous nonlocking suture including decidua. In DLT the first layer comprised a continuous nonlocking suture including decidua, followed by a second continuous, nonlocking suture (Figure 1). In both techniques, we used 0/0 polyglactin 910 suturing material with a blunt needle (Vicryl, Ethicon^®^, Diegem, Belgium). Additional hemostasis sutures could be laid regardless of the closure method. All obstetricians who performed the surgery had similar experience, and those with less training were always supervised by a senior attending physician.

**Figure 2:**
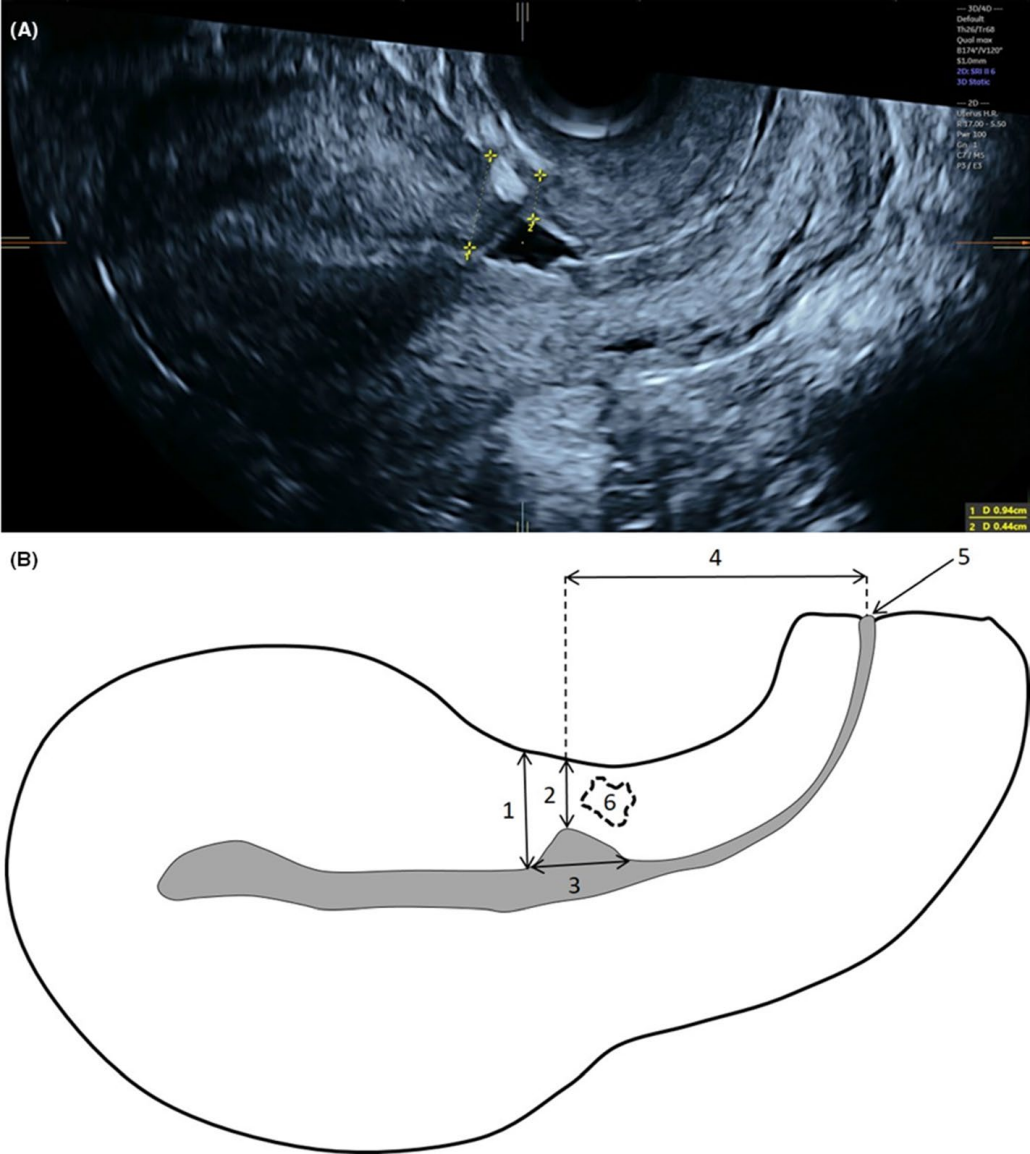
(A) Transvaginal ultrasound demonstrating measurement of total myometrial thickness (1) and residual myometrial thickness (2). (B) Schematic diagram showing CS scar placement and dimensions measurement: total myometrial thickness (1), residual myometrial thickness (2), width of the scar defect (3), distance between the scar and the external cervical ostium (4), external cervical ostium (5), myometrial defects without contact with the uterine cavity (6) [Color figure can be viewed at http://wileyonlinelibrary.com]

During the follow‐up control visits at 6 weeks, 6 and 12 months, all patients underwent a three‐dimensional transvaginal ultrasound of the uterus using a GE Voluson E8 Expert ultrasound system (General Electric, Zipf, Austria) equipped with a 2.8‐10 MHz transvaginal probe in the lithotomy position with an empty urinary bladder. Imaging was performed in the mid‐sagittal plane with the angle of acquisition set at 120°. Two volume datasets in longitudinal and transverse sections were acquired and stored for later analysis using the software 4d view (General Electric Medical Kretz technik, Zipf, Austria). Data analysis were undertaken by two of the authors (J.H. and L.K.), blinded to clinical data. The following sonographic features were assessed: position of the uterus (anteflexion or retroflexion), visibility of the CS scar, presence of a scar defect (yes or no); any visible defect in the scar was classified as a defect. The following scar measurements were taken: total myometrial thickness, residual myometrial thickness (RMT), scar width, the distance between the scar and the external cervical os. We also noted myometrial defects with or without contact with the uterine cavity (scar defect character) (Figure 2).

Primary outcome was the mean RMT related to the SLT or DLT. Secondary outcomes included: frequency of uterine defects, the position of the uterus, frequency of uterine defects with respect to the position of the uterus, position of the scar and frequency of defects with respect to the stage of labor in which the CS was performed. We recorded the incidence of selected postoperative complications at 6 weeks postpartum.

Statistical analyses were performed using SPSS software version 13.0 (IBM Corp., Armonk, NY, USA). Participant baseline and ultrasound characteristics were presented. The SLT and DLT group were compared. For continuous, normally distributed variables, we used Student's *t* test. The nonparametric tests (Wilcoxon‐Mann‐Whitney test) were used for continuous, non‐normally distributed variables. To test symmetry in the contingency table with dichotomous variables we used Fisher's exact test. A *P* value <0.05 was considered to be significant. Apart from simple tests, linear model (two‐way ANOVA) was carried out to examine the effect of vaginal findings during indication for CS on the scar. To test the development of categorized variables (including dichotomous variables) over time and dependence on suturing techniques, the generalized linear mixed model was used.

### 2.1 Ethical approval

The design of this study was approved by the institutional ethics committee (ethics committee number 3/2010).

## 3 RESULTS

**Figure 3:**
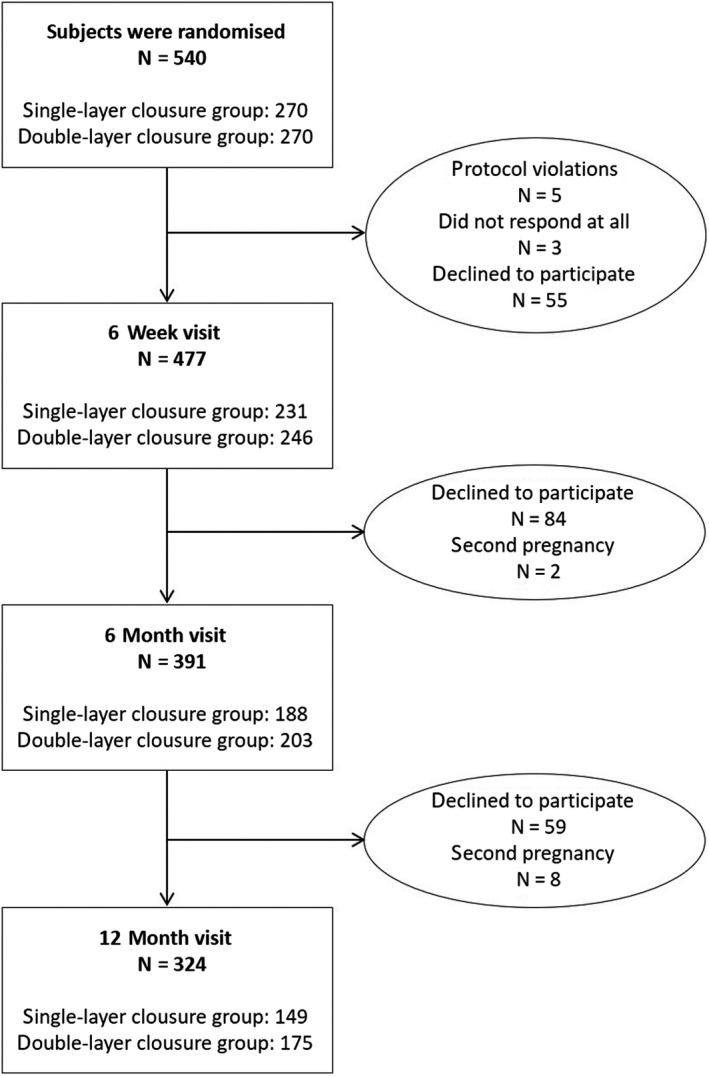
Flow chart summarizing selection of participants who underwent single‐ or double‐layer uterine suture technique. We observed five protocol violations

A total of 540 pregnant women (270 in the SLT, 270 in the DLT) were included in the study. Drop‐out rate was 216 cases (40%) (Figure 3). Table 1 summarizes the basic demographics and obstetric data of the 12‐month visit cohort and those who dropped out. Six weeks postpartum, there were no differences in uterine position, presence or localization of the defect and scar sonomorphology. In all, 324 women attended the 12‐month visit, 149 of whom underwent SLT and 175 DLT. Their demographic and obstetric data did not differ, nor did the incidence of selected complications (Table 2). Repeated observational data on uterine position, presence and type of defects with missing cases are presented in Tables 3, 4, 5, 6. The CS scar measurements at 12 months postpartum are listed in Table 7. Defects in the SLT group were significantly (0.002) wider (4.8 vs 4.0 mm). The RMT in the SLT group were significantly (0.019) thinner (4.6 vs 5.2 mm). At 12 months there were no significant difference in the total myometrial thickness (0.777). One year postpartum, defects in the DLT group were significantly (0.002) closer to the external cervical os (30.0 vs 33.0 mm). Women who underwent CS at the stage of full cervical dilation had scars that were closer to the external cervical os (0.000), RMT was thinner (0.010) and width of the defect smaller (0.001). Other ultrasound scar measurements were not influenced by the vaginal finding at the time of the CS indication.

**Table 1:** Demographics and obstetric data comparison of study subjects who attended the 12‐month visit and women who dropped out. Ultrasound outcomes comparison of study subjects attending the 12‐month visit and women who dropped out after the 6‐week check‐up

Parameter	Follow up		*P*
	12‐month visit (n = 324)	Drop out (n = 216)	
Demographic and obstetrics details			
Maternal age (y)	31.7 ± 3.8	31.1 ± 4.2	0.139^b^
BMI (kg/m^2^)	22.9 (20.2‐24.8)	23.1 (20.5‐27.8)	0.925^c^
Gestational age at delivery (gestational weeks)	40.2 (39‐41)	40.2 (39‐41)	0.928^c^
Previous surgery on the uterus	43 (13.3)	23 (15.0)	0.612^d^
Assisted reproduction: IVF/ICSI	21 (6.5)	9 (5.9)	0.489^d^
Gestational diabetes	12 (3.7)	7 (4.6)	0.409^d^
Hypertensive disorders	11 (3.4)	4 (2.8)	0.443^d^
Type of cesarean section			
Acute in pregnancy	15 (4.6)	2 (0.9)	0.062^d^
Acute during labor	132 (40.7)	92 (42.6)	
Planned in pregnancy	39 (12.0)	29 (13.4)	
Planned during labor	138 (42.6)	93 (43.1)	
Hysterotomy closure			
Single‐layer	149 (46.0)	100 (46.3)	0.167^d^
Double‐layer	175 (54.0)	116 (53.7)	
Cervical dilation			
No dilation	91 (28.1)	54 (25.0)	0.360^d^
Partial dilation	166 (51.2)	125 (57.9)	
Full dilation	67 (20.7)	37 (17.1)	
	6‐week control (n = 153)^a^		
Uterine position			
Anteflexion	176 (54.3)	88 (56.1)	0.570^d^
Retroflexion	148 (45.7)	65 (42.5)	
CS scar description			
Intact scar	62 (19.1)	38 (24.8)	0.199^d^
Presence of scar defect	262 (80.9)	115 (75.2)	
Scar measurements and localization			
Total myometrial thickness (mm)	11.5 (9.7‐13.0)	10.5 (9.2‐12.1)	0.115^c^
Residual myometrial thickness (mm)	5.8 ± 2.2	5.6 ± 1.9	0.340^b^
Width of the defect (mm)	4.3 (2.9‐5.9)	4.2 (2.8‐5.8)	0.180^c^
Scar‐external cervical os distance (mm)	31.0 (27.0‐35.0)	33.0 (28.0‐35.4)	0.211^c^

Abbreviations: CS, cesarean section; IVF/ICSI, in vitro fertilization/intracytoplasmatic sperm injection.

a The 63 women who did not attend the 6‐week check‐up were not included. Characteristics are presented as mean ± SD for normally distributed variables, median and interquartile range for non‐normally distributed variables. Categorical variables were presented as total number (percentage in group).

b Student′s *t* test.

c Wilcoxon‐Mann‐Whitney test.

d Pearson Chi‐square test.

**Table 2:** Demographics, obstetric data and selected complications at 6 week postpartum of study subjects who finished the 12‐month follow up. Characteristics are presented as median and interquartile range for non‐normally distributed variables. Categorical variables are presented as total number (percentage in group)

Parameter	Closure technique		*P*
	Single‐layer (n = 149)	Double‐layer (n = 175)	
Demographic and obstetrics details			
Maternal age (y)	31.0 (29.0‐34.0)	32.0 (29.0‐34.0)	0.392^a^
BMI (kg/m^2^)	22.4 (20.4‐25.3)	22.3 (20.1‐24.2)	0.602^a^
Gestational age at delivery (gestational weeks)	40.0 (39.0‐41.0)	40.0 (40.0‐41.0)	0.446^a^
Type of cesarean section			
Acute in pregnancy	8 (5.4)	8 (4.6)	0.850^b^
Acute during labor	62 (41.6)	70 (40.0)	
Planned in pregnancy	16 (10.7)	22 (12.6)	
Planned during labor	63 (42.3)	75 (42.9)	
Cervical dilation			
No dilation	42 (28.2)	49 (28.0)	0.245^b^
Partial dilation	82 (55.0)	84 (48.0)	
Full dilation	25 (16.8)	42 (24.0)	
6‐week complications			
None	135 (90.6)	159 (90.9)	0.263^b^
Maternal infectious morbidity	7 (4.7)	7 (4.0)	
Operative procedures on wound	4 (2.7)	1 (0.6)	
Other (placental remnants, transfusion)	3 (2.0)	8 (4.6)	

a Wilcoxon‐Mann‐Whitney test.

b Pearson Chi‐square test.

**Table 3:** Repeated observation data on *presence of scar defects* with missing cases. Categorical variables are presented as total number (percentage in group)

Follow up	Closure technique			
	Single‐layer (n = 149)		Double‐layer (n = 175)	
	Presence of scar defect			
	Yes	No	Yes	No
6 weeks^a^	187 (81.0)	44 (19.0)	191 (77.6)	55 (22.4)
6 months^b^	148 (78.7)	40 (21.3)	155 (76.4)	48 (23.6)
12 months^c^	124 (83.2)	25 (16.8)	127 (72.6)	48 (27.4)

a Total number of women who attended the 6‐week follow up = 477.

b Total number of women who attended the 6‐month follow up = 391.

c Total number of women who attended the 12‐month follow up = 324.

**Table 4:** Repeated observation data on *uterine position* with missing cases. Categorical variables are presented as total number (percentage in group)

Follow up	Closure technique			
	Single‐layer (n = 149)		Double‐layer (n = 175)	
	Uterine position			
	Anteflexion	Retroflexion	Anteflexion	Retroflexion
6 weeks^a^	122 (52.8)	109 (47.2)	141 (57.3)	105 (42.7)
6 months^b^	123 (65.4)	65 (34.6)	151 (74.4)	52 (25.6)
12 months^c^	90 (60.4)	59 (39.6)	125 (71.4)	50 (28.6)

a Total number of women who attended the 6‐week follow up = 477.

b Total number of women who attended the 6‐month follow up = 391.

c Total number of women who attended the 12‐month follow up = 324.

**Table 5:** Repeated observation data on s*car defects in anteflexion/retroflexion* (*AVF/RVF) uterus* with missing cases. Categorical variables are presented as total number (percentage in group)

Follow up	Closure technique			
	Single‐layer (n = 149)		Double‐layer (n = 175)	
	Scar defect in AVF uterus			
	Yes	No	Yes	No
6 weeks^a^	105 (86.1)	17 (13.9)	116 (82.3)	25 (17.7)
6 months^b^	102 (82.9)	21 (17.1)	117 (77.5)	34 (22.5)
12 months^c^	81 (90.0)	9 (10.0)	90 (72.0)	35 (28.2)

Follow up	Scar defect in RVF uterus			
	Yes	No	Yes	No
6 weeks^a^	82 (75.2)	27 (24.8)	75 (71.4)	30 (28.6)
6 months^b^	46 (70.8)	19 (29.2)	38 (73.1)	14 (26.9)
12 months^c^	43 (72.9)	16 (27.1)	37 (74.0)	13 (26.0)

a Total number of women who attended the 6‐week follow up = 477.

b Total number of women who attended the 6‐month follow up = 391.

c Total number of women who attended the 12‐month follow up = 324.

**Table 6:** Repeated observation data on s*car defect presence and defect contact with the uterine cavity* with missing cases. Categorical variables are presented as total number (percentage in group)

Follow up	Closure technique					
	Single‐layer (n = 149)			Double‐layer (n = 175)		
	Presence and defect contact with the uterine cavity					
	Yes	No	Normal scar	Yes	No	Normal scar
6 weeks^a^	111 (48.1)	76 (32.9)	44 (19.0)	118 (48.0)	73 (29.7)	55 (22.4)
6 months^b^	112 (59.6)	36 (19.1)	40 (21.3)	114 (56.2)	41 (20.2)	48 (23.6)
12 months^c^	84 (56.4)	40 (26.8)	25 (16.8)	92 (52.6)	35 (20.0)	48 (27.4)

Notes

Yes: scar defect is present and is in contact with the cavity; No: scar defect is present and is not in contact with the cavity, normal scar defect is not present.

a Total number of women who attended the 6‐week follow up = 477.

b Total number of women who attended the 6‐month follow up = 391.

c Total number of women who attended the 12‐month follow up = 324.

**Table 7:** Sonographic cesarean section (CS) scar measurement and localization at 12‐month follow up and relation to cervical dilation at time of delivery. Characteristics are presented as mean ± SD for normally distributed variables, median and interquartile range for non‐normally distributed variables

Parameter	Closure technique		*P*
	Single‐layer (n = 149)	Double‐layer (n = 175)	
CS scar description			
Scar measurements and localization			
Total myometrial thickness (mm)	10.0 (8.5‐11.5)	10.0 (8.6‐11.7)	0.777^b^
Residual myometrial thickness (mm)	4.6 (±1.9)	5.2 (±2.2)	0.019^a^
Width of the defect (mm)	4.8 (3.2‐6.6)	4.0 (3.0‐5.4)	0.002^b^
Scar‐external cervical os distance (mm)	33.0 (29.0‐37.0)	30.0 (25.7‐34.7)	0.002^b^
Residual myometrial thicknes <2.5 mm	18 (12.2)	12 (6.8)	0.019^a^
CS scar description and relation to vaginal finding at CS indication			
Total myometrial thickness			
No cervical dilation	10.5 (±2.6)	10.1 (±2.0)	0.533^c^
Partial cervical dilation	10.2 (±2.1)	10.2 (±1.8)	
Full cervical dilation	8.8 (±2.4)	9.9 (±2.4)	
Residual myometrial thickness			
No cervical dilation	4.8 (±1.7)	5.7 (±2.1)	0.010^c^
Partial cervical dilation	4.7 (±2.0)	5.0 (±1.9)	
Full cervical dilation	4.3 (±2.2)	5.0 (±2.5)	
Width of the defect			
No cervical dilation	5.6 (±2.8)	4.3 (±2.1)	0.001^c^
Partial cervical dilation	4.7 (3.2‐6.0)	4.0 (3.2‐5.5)	
Full cervical dilation	4.0 (2.9‐7.3)	4.0 (3.1‐5.3)	
Scar‐external cervical os distance			
No cervical dilation	35.5 (29.3‐37.0)	33.0 (29.0‐39.0)	0.000^c^
Partial cervical dilation	33.6 (±6.1)	30.1 (±5.5)	
Full cervical dilation	28.9 (±9.0)	27.0 (±5.8)	

Student′s *t* test.

Wilcoxon‐Mann‐Whitney test.

Two‐way ANOVA (with interaction).

Longitudinal observational data on uterine position and presence and type of defects in this cohort are presented in Tables 8, 9, 10, 11. The incidence of scar defects was not statistically significant between controls. The difference between groups with different suturing methods was statistically significant (0.036). A higher proportion of the defects were seen in the SLT group.The position of the uterus varied greatly between controls (0.000), especially between 6‐week and 6‐month controls. The difference between the 6‐ and 12‐month controls was already statistically insignificant. The difference between groups with different suturing methods was not statistically significant. The combination of uterine position and scar defect presence changed significantly between controls (0.001) and it varied significantly depending on the suturing method (0.003). A higher incidence of uterine position in retroflexion and defects was seen in the 6‐week control in the SLT group. Defects with or without contact with the uterine cavity changed statistically between controls (0.017). Both types of defects occurred more frequently in the 6‐week follow up than in stage II and III controls. The difference based on suturing method is at the limit of statistical significance (0.065). Both types of defects are more common in the SLT group. For any of the variables uterine position, scar defect, combination of both and scar placement, the difference between groups according to the suturing method did not change statistically significantly over time (interaction of time and suture type is not significant).

**Table 8:** Longitudinal observation data on *Presence of scar defect* in women with all three follow up controls (total number of women who attended the 12‐month follow up, with all three controls = 324). Test of dependence of outcome on time (generalized linear mixed model): test significance of variable *Time* is 0.247, test significance of variable *Closure technique* is 0.036 and test significance of interaction *Time* with *Closure technique* (change of closure technique outcome in time) is 0.370

Follow up	Closure technique			
	Single‐layer (n = 149)		Double‐layer (n = 175)	
	Presence of scar defect			
	Yes	No	Yes	No
6 weeks	123 (82.6)	26 (17.4)	140 (80.0)	35 (20.0)
6 months	118 (79.2)	31 (20.8)	129 (73.7)	46 (26.3)
12 months	124 (83.2)	25 (16.8)	127 (72.6)	48 (27.4)

**Table 9:** Longitudinal observation data on *Uterine position* in women with all three follow‐up controls (total number of women who attended 12‐month follow up, with all three controls = 324). Test of dependence of outcome on time (generalized linear mixed model): test significance of variable *Time* is 0.000, test significance of variable *Closure technique* is 0.132 and test significance of interaction *Time* with *Closure technique* (change of closure technique outcome in time) is 0.270

Follow up	Closure technique			
	Single‐layer (n = 149)		Double‐layer (n = 175)	
	Uterine position			
	Anteflexion	Retroflexion	Anteflexion	Retroflexion
6 weeks	78 (52.3)	71 (47.7)	97 (55.4)	78 (46.6)
6 months	97 (65.1)	52 (34.9)	128 (73.1)	47 (26.9)
12 months	90 (60.4)	59 (39.6)	125 (71.4)	50 (28.6)

**Table 10:** Longitudinal observation data on *Scar defect in anteflexion/retroflexion (AVF/RVF) uterus* in women with all three follow‐up controls (total number of women who attended 12‐months follow up, with all three controls = 324). Test of dependence of outcome on time (generalized linear mixed model): test significance of variable *Time* is 0.001, test significance of variable *Closure technique* is 0.003 and test significance of interaction *Time* with *Closure technique* (change of closure technique outcome in time) is 0.821

Follow up	Closure technique			
	Single‐layer (n = 149)		Double‐layer (n = 175)	
	Scar defect in AVF uterus			
	Yes	No	Yes	No
6 weeks	70 (89.7)	8 (10.3)	80 (82.5)	17 (17.5)
6 months	81 (83.5)	16 (16.5)	94 (73.4)	34 (26.6)
12 months	81 (90.0)	9 (10.0)	90 (72.0)	35 (28.2)

Follow up	Scar defect in RVF uterus			
	Yes	No	Yes	No
6 weeks	53 (74.6)	18 (25.4)	60 (76.9)	18 (23.1)
6 months	37 (71.2)	15 (28.8)	35 (74.4)	12 (25.5)
12 months	43 (72.9)	16 (27.1)	37 (74.0)	13 (26.0)

**Table 11:** Longitudinal observation data on *Scar defect presence and defect contact with the uterine cavity* in women with all three follow‐up controls (total number of women who attended 12‐month follow up, with all three controls = 324). Test of dependence of outcome on time (generalized linear mixed model): test significance of variable *Time* is 0.017, test significance of variable *Closure technique* is 0.065 and test significance of interaction *Time* with *Closure technique* (change of closure technique outcome in time) is 0.493

Follow up	Closure technique					
	Single‐layer (n = 149)			Double‐layer (n = 175)		
	Presence and defect contact with the uterine cavity					
	Yes	No	Normal scar	Yes	No	Normal scar
6 weeks	75 (50.3)	48 (32.2)	26 (17.4)	87 (49.7)	53 (30.3)	35 (20.0)
6 months	91 (61.1)	27 (18.1)	31 (20.8)	93 (53.1)	36 (20.6)	46 (26.3)
12 months	84 (56.4)	40 (26.8)	25 (16.8)	92 (52.6)	35 (20.0)	48 (27.4)

## 4 DISCUSSION

The prevalence of scar defects on transvaginal ultrasound in our study was 83.2% in the SLT and 72.6% in the DLT group at the 12‐month follow up. We observed a higher prevalence of defects compared with other groups after first CS (37‐61%).^3^, ^9^, ^10^, ^12^ The discrepancy is most likely to be explained not only by the different definitions of defect that have been used in different studies, but also by including defects that were not in contact with the uterine cavity. The 12‐month prevalence of more serious defects (RMT <2.5 mm)^9^, ^13^, ^14^ in contact with the cavity was 12.2% in the SLT and 6.8% in the DLT group.

We show that the presence of a defect and the scar position are relatively stable during the first postpartum year but that their appearance changes. Defects with or without contact with the uterine cavity changed statistically between controls. Both types of defects occurred more frequently in the 6‐week follow up and were more common in the SLT group. The most notable example was the defect without contact with the uterine cavity, which represented 30% of scar defects at 6 weeks and 20% at 6 months. During healing, this structure can disappear and can be changed into defects with contact with the cavity. Healing is a long‐term, ongoing process of tissue remodeling, peaking in intensity within 6 months after the primary insult.^15^, ^16^ On the basis our data we assume that CS scar healing should be completed after 6 months. This is consistent with previous studies.^14^

The most important decision is whether to use an SLT or DLT to improve the scar quality and decrease the risk of uterine rupture and dehiscence in the subsequent pregnancy. Data from Swedish registers demonstrate no significant difference in the rate of uterine rupture.^17^ An earlier meta‐analysis including retrospective and prospective studies reported that locked SLT, compared with DLT, is associated with a fourfold increase in the risk of uterine rupture.^18^ We do not have any clinical data from subsequent pregnancies in our patients and are thus unable to confirm this finding.

We have shown that DLT is associated with smaller defects and with thicker RMT. This is in contrast to evidence based on randomized trials which does not support a specific type of uterine closure. SLT and locked first layer are possibly associated with thinner residual RMT.^19^

Prospective studies using transvaginal ultrasound of the scar, favor an unlocked suture with exclusion of the decidua to optimize the placement of the muscle layers and their regeneration.^19^, ^20^ Roberge et al demonstrate in a randomized controlled trial that DLT with a first unlocked layer excluding the decidua, compared with locked SLT including the decidua, is associated with a greater RMT and healing ratio.^21^ But the lack of statistical power did not allow the authors to draw a definitive conclusion. It is possible that excluding the decidua from the first suture induces a better adaptation of myometrium. We have included decidua in the first suture and thus are unable to confirm this suggestion.

The reason for the more distal scars and defects in women with CS at full dilation is clear. At stage II of labor, the cervix creates a continuous birth canal with the uterine cavity and is pulled up. Therefore, the incision is finally located caudal to the external cervical ostium.

In contrast to other studies, we were not able to prove that scars with larger defects reside more caudally than intact scars or scars with smaller defects.^22^ But those findings were influenced by cases with more than one CS.

We have shown a change in uterine position between 6 weeks and 1 year from retroflexion to anteflexion, with no relation to closure technique. We do not have data on its position before the pregnancy and thus were unable to demonstrate that the CS and the closure technique affected it. The reason for change in uterine position is unknown: it could be due to tissue healing and scar remodeling, but it may also interfere with healing and tissue extension. Vikhareva Osser et al observed more scar defects in women with a uterus in retroflexion.^9^ A higher incidence of uterine position in retroflexion and defects was only seen in our study in the 6‐week control in the SLT group.

Our study is not limited only to CS performed in women before or in early labor. Women in advanced labor were also included. The women in both groups were selected from the same caucasian community and the comparability between the two groups was high. The scar was longitudinally evaluated by two independent observers blinded to the treatment allocation in a population having a primary CS. Another strength of our study was the uniform use of a specific suture method for both closures. On the other hand, cervical dilation, duration of labor or oxytocin augmentation are factors that increase the risk of a large scar defect in non‐pregnant women.^23^

The main limitation of the study is the drop‐out rate. We did not examine further why women discontinued the follow up. Another limitation is that RMT represents an indirect evaluation of scar healing and demonstrates a surrogate outcome for the prediction of negative consequences. On the other hand, there are enough data showing that the presence of scar defects and RMT value are correlated with such adverse events.^10^, ^23^ Also, we did not perform an ultrasonographic examination according to menstrual cycle, as recommended by another group.^7^ Synchronization was not possible because many women were breastfeeding and had secondary amenorrhea. This study was primarily an urogynecological research targeted at the influence of the first pregnancy and delivery on female pelvic floor; therefore this subanalysis, which is focused on scar assessment after CS, was not registered as a randomized control trial.

## 5 CONCLUSION

Our data demonstrate the benefit of DLT. Defects in SLT group were more common, wider and had thinner RMT. Most changes in the scar area occurred during the first 6 months. Although recent discussion has focused mainly on the number of suture layers, the current knowledge highlights the importance of decidua suture exclusion.

## CONFLICT OF INTERESTS

The authors have stated explicitly that there are no conflicts of interest in connection with this article. This study was supported by PROGRES Q 34, Charles University project, Prague, Czech Republic.
